# Hyponatremia Following Endoscopic Third Ventriculostomy in an Adolescent with an Aqueductal Web: A Case Report

**DOI:** 10.3390/reports9020122

**Published:** 2026-04-17

**Authors:** Tingting Feng, Lee Ping Ng, Wan Tew Seow, Sharon Y. Y. Low

**Affiliations:** 1 Neurosurgical Service, KK Women’s and Children’s Hospital, 100 Bukit Timah Road, Singapore 229899, Singapore; 2 Department of Neurosurgery, National Neuroscience Institute, 11 Jalan Tan Tock Seng, Singapore 308433, Singapore; 3 SingHealth Duke-NUS Neuroscience Academic Clinical Program, 11 Jalan Tan Tock Seng, Singapore 308433, Singapore; 4 SingHealth Duke-NUS Pediatrics Academic Clinical Program, 100 Bukit Timah Road, Singapore 229899, Singapore

**Keywords:** aqueductal web, endoscopic third ventriculostomy, hydrocephalus, hyponatremia, syndrome of inappropriate antidiuretic hormone secretion

## Abstract

Background and Clinical Significance: Endoscopic third ventriculostomy (ETV) is a well-established cerebrospinal fluid (CSF) diversion technique for treating obstructive hydrocephalus. Here, the complication of post-ETV hyponatremia is rare. Separately, aqueductal web as a cause of obstructive hydrocephalus is also an uncommon occurrence. We present an unusual case of an adolescent who presented with late symptoms of obstructive hydrocephalus secondary to an aqueductal web and developed a delayed onset of post-operative hyponatremia after a successful ETV procedure. Pertinent aspects of the case are discussed in corroboration with the recent literature. Case Presentation: A previously well 14 year old presented with symptoms of raised intracranial pressure. Neuroimaging demonstrated progressively enlarging ventricles associated with an aqueductal web. She underwent an uneventful ETV and was discharged home. However, she was readmitted for symptomatic hyponatremia that was investigated and most likely attributed to Syndrome of Inappropriate Antidiuretic Hormone Secretion (SIADH). She was managed with fluid restriction with good clinical improvement. Conclusions: We herein report a case of delayed onset of obstructive hydrocephalus secondary to an aqueductal web, treatment challenges faced and the patient’s unexpected occurrence of hyponatremia after a technically successful ETV. This emphasizes that clinicians need to be mindful of this potential post-operative complication and the ability to discern subtle symptoms in a patient whose clinical signs may not be straightforward.

## 1. Introduction and Clinical Significance

Endoscopic third ventriculostomy (ETV) is an established cerebrospinal fluid (CSF) diversion technique in hydrocephalus. This minimally invasive procedure serves to divert CSF flow from the third ventricle directly into the basal cisterns, hence circumventing the cerebral aqueduct and fourth ventricle. The main advantages of ETV include utilizing the patient’s own CSF pathways to cope with the excess fluid and concurrent avoidance of implanting a permanent ventriculoperitoneal shunt (VPS). Despite its purported advantages, ETV procedures are associated with established complications of iatrogenic neurovascular damage, intracranial bleeding, infection and neurological deficit. Less common side effects include hormonal dysfunction secondary to injury of the hypothalamic-pituitary axis. Specifically, iatrogenic hyponatremia secondary to Syndrome of Inappropriate Antidiuretic Hormone (SIADH) secretion is rare and unusual.

Separately, the etiologies underlying pediatric hydrocephalus are diverse and heterogeneous [1]. More common causes include congenital aqueductal stenosis, neural tube defects and intracranial tumors [2,3]. In contrast, aqueductal webs resulting in obstructive hydrocephalus are infrequent [4]. These lesions are postulated to be caused by ependymal inflammation that leads to scarring and obstruction of the flow route of CSF in the aqueduct [5]. We describe a unique case of an adolescent who presented with late symptoms of obstructive hydrocephalus secondary to an aqueductal web and developed a delayed onset of post-operative hyponatremia after a successful ETV procedure. Pertinent aspects of the case are discussed in corroboration with the existing literature.

## 2. Case Presentation

**Figure 1:**
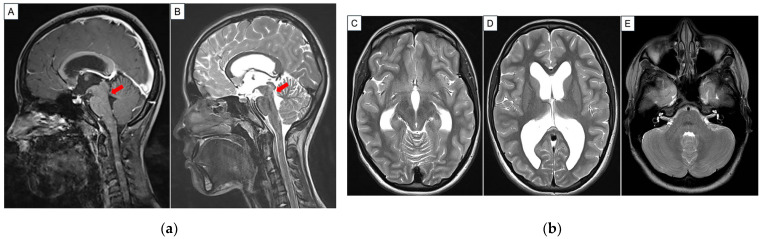
(**a**) Representative post-contrast T1-weighted [Image (**A**)] and T2-weighted [Image (**B**)] sequences of the patient’s MRI brain image in the sagittal direction. The red arrow in both images points to a tissue web within the aqueduct of Sylvius. (**b**) Representative T2-weighted sequence MRI brain images the in axial direction depicting [Image (**C**)] slightly enlarged third ventricle and bilateral temporal horns; [Image (**D**)] bilaterally enlarged frontal and posterior ventricles and [Image (**E**)] no enlargement of the fourth ventricle. Of note, there is no significant transependymal CSF flow despite the ventricular enlargement.

A 14-year-old female presented with low-grade but persistent headaches after a minor head injury. She had a childhood history of streptococcal septicemia that required ventilatory support at the age of 4 years. However, she recovered completely from the infection. Otherwise, there was no other significant past medical history. Clinical examination showed an alert and cooperative individual with full Glasgow Coma Scale (15 out of 15) and no neurological deficit. She did not have visual disturbance, gait disturbance or neuro-cognitive concerns. In addition, there were no symptoms of raised intracranial pressure. The remainder of her systems review was unremarkable. As part of the work-up, a magnetic resonance imaging (MRI) brain scan was arranged. This reported mildly dilated lateral and third ventricles with isointense membrane-like structures in the aqueduct of Sylvius, suggestive of aqueductal webs causing stenosis (Figure 1). A formal ophthalmology examination did not show evidence of papilledema. Put together, the impression was that her headaches were more likely concussion-related, and the MRI findings were incidental. She improved with a trial of conservative management with rest, analgesia and physiotherapy. Pertaining to her neuroimaging results, a decision was made for close outpatient surveillance, with a view to perform intervention if necessary. Subsequently, the patient continued to remain well without any new neurological symptoms.

**Figure 2:**
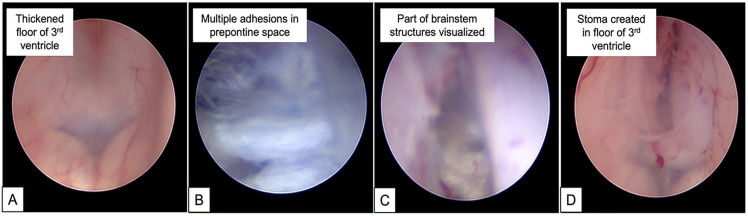
Consecutive intraoperative photos taken during neuroendoscopy: (**A**) Prior to ETV, the floor of the third ventricle is noted to be mostly opaque and thickened. (**B**) Multiple adhesions in the prepontine space encountered after the floor of the third ventricle was opened. (**C**) Post-adhesiolysis, there was improved visualization of structures (**D**) Stoma created in the floor of the third ventricle after ETV. Here, the thickened edges of the stoma reflect the technical challenges faced during the procedure. Nonetheless, the stoma edges were noted to be flapping well—an indication of good CSF flow through the ventriculostomy.

Approximately 12 months later, she started to complain of worsening headaches associated with intermittent visual blurring. Otherwise, she was able to continue her daily activities of living without issues. A repeat ophthalmology review was arranged. This time, it was demonstrated that she had bilateral papilledema. Her visual acuity, pupillary reactivity, color vision and visual fields were within the normal range. A repeat MRI brain did not demonstrate significant increase in the size of her ventricles in comparison to her previous scans (Figure 2). In view of her progressive symptoms and existing neuroimaging results, a working diagnosis of symptomatic obstructive hydrocephalus secondary to aqueductal webs was made. She was recommended to undergo a CSF diversion procedure to treat the potentially life-threatening raised intracranial pressure (ICP). Therapeutic options of ETV versus a ventriculoperitoneal shunt (VPS) were discussed at length with the patient and her caregivers. In view of her favorable ETVSS score and long-term concerns related to VPS malfunction and failure, they opted for an ETV procedure [1,6,7,8].

The patient underwent an ETV for which the technical details have been previously described [8]. For this case, frameless stereotactic neuro-navigation (Stealth Station S8; Medtronic, Inc., Minneapolis, MN, USA) was used to plan an optimal trajectory into the third ventricle. Under asepsis, a right pre-coronal burrhole in the mid-pupillary line was made with the patient in a supine position. Next, a rigid zero-degree Little LOTTA^®^ neuroendoscope (Storz, Tuttlingen, Germany) was introduced transcortically into the frontal horn of the lateral ventricle [8]. Intraoperatively, the CSF was clear and colorless but noted to be under high pressure upon cannulation into the ventricle. Of note, the floor of the third ventricle was opaque and thickened. A stoma was created in the third ventricle floor between mammillary bodies and the infundibular recess using the Decq forceps and gently widened with a 3-French-sized Fogarty catheter balloon. Upon opening the third ventricular floor, the basilar artery and the Liliequist membrane were noted to be obscured by multiple web-like adhesions. A decision was made to start the adhesiolysis in the anterior prepontine space due to the risks of iatrogenic injury to adjacent structures. At the end of the procedure, adequate CSF flow through the created ETV stoma was visualized (Figure 2).

**Figure 3:**
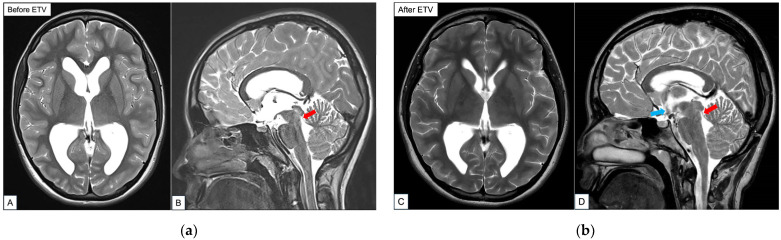
Representative T2-weighted sequence MRI brain images before ETV (**a**) versus after ETV (**b**). An objective radiological measurement of the ventricle size using the axial image is calculated via the Evans Index [9]. Briefly, this is the ratio of the maximum width of the frontal horns of the lateral ventricles and the maximal internal diameter of the skull at the same level [9,10]. (**a**) Pre-operative MRI brain images in axial (Image (**A**)) and sagittal (Image (**B**)) directions, depicting enlarged ventricles whereby the calculated Evans Index is 0.38. Red arrow points to a tissue web within the aqueduct of Sylvius. (**b**) Post-operative MRI brain images in axial (Image (**C**)) and sagittal (Image (**D**)) directions that show noticeably smaller ventricles in comparison to (**a**). In Image (**D**), there is a flow void seen (blue arrow). Here, the calculated Evans Index is 0.28. The red arrow points to a tissue web within the aqueduct of Sylvius.

An MRI brain scan performed 48 h after surgery confirmed the presence of a flow void in the floor of the third ventricle into the prepontine cistern. Additionally, the intraparenchymal sulcal spaces were noted to be the more open and there was a reduction in the size of bilateral ventricles (Figure 3). The results of laboratory investigations performed on the sampled CSF were negative for infection, cytology or profile anomalies. Owing to concerns of brain manipulation during the challenging ETV procedure, a short course of oral dexamethasone to be weaned off over 7 days was initiated. Overall, the patient’s immediate post-operative period was uneventful. She remained clinically well and was discharged home 3 days after her surgery.

**Figure 4:**
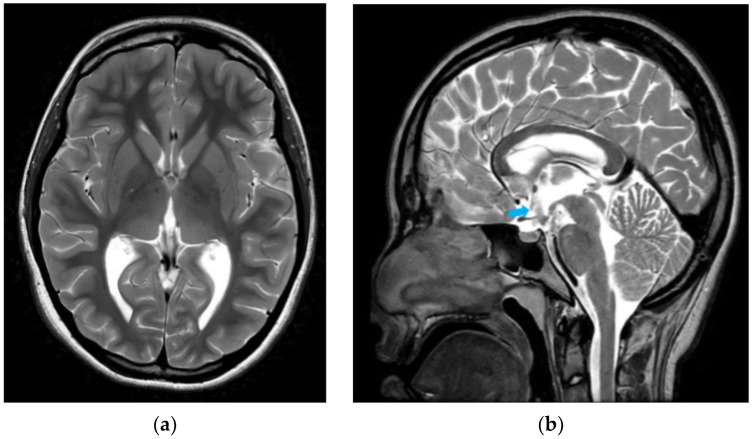
Representative T2-weighted sequence MRI brain images 6 months after ETV in axial (**a**) and sagittal (**b**) views, respectively. (**a**) In this MRI image in the axial direction, the ventricle size remains reduced in comparison to before surgery. (**b**) In the corresponding sagittal view of the same MRI study, there is radiological evidence of a flow void (blue arrow) into the prepontine cistern.

**Figure 5:**
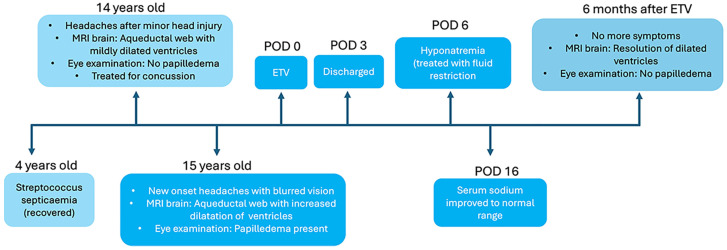
Schematic timeline diagram of patient’s featured clinical conditions.

However, the patient returned on post-operative day 6 with severe bifrontal headaches, nausea, lethargy and generalized discomfort. Her caregivers also reported that she had onset of unusual emotional lability. There were no new fevers, seizures or focal neurological deficits. Clinical examination demonstrated an alert, cooperative patient with a full Glasgow Coma Scale. She had good skin turgor, moist mucous membranes and no orthostatic hypotension—indicating euvolemia—and was able to drink to thirst. Her operative wound was clean, dry and intact. Further examination did not demonstrate a palpable neck mass, dysphagia or voice changes. An urgent computed tomographic (CT) brain scan was arranged to exclude ETV failure and/or other intracranial pathology. However, there was no new bleed or worsening hydrocephalus. Ophthalmological examination demonstrated bilaterally clear, conjunctivae and her previous papilledema had improved. Blood investigations were otherwise unremarkable except for her serum sodium, which was very low at 121 mmol/L (normal range 138 to 145 mmol/L). Further tests to work up her hyponatremia suggested a likely presumptive diagnosis of SIADH, despite a normal urinary sodium result (Table 1). Serum cortisol was not included because the patient was still on her final low doses of oral dexamethasone at this time. Based on the clinical history, physical examination and investigations, other causes of hyponatremia were excluded. Overall, the patient had pathophysiology more consistent with water retention, rather than natriuretic volume loss. As recommended by the pediatric endocrinologist, she was placed on a trial of fluid restriction under close monitoring which successfully restored her serum sodium levels to normal. Her previous symptoms of headaches, gastrointestinal concerns and behavioral changes also simultaneously resolved. The patient was subsequently discharged. At 6 months post-surgery, a repeat MRI scan showed that her ventricle sizes were still reduced and the ETV stoma was patent (Figure 4). Additionally, she was noted to be clinically well and had resumed normal activities. Figure 5 provides a summary timeline of her clinical conditions.

## 3. Discussion

### 3.1. Overview of Case Description

We highlight two unique aspects of our case: firstly, the late onset of hydrocephalus due to an aqueductal web and next, the development of post-operative hyponatremia. Broadly speaking, an aqueductal web is a membrane composed of fibrillary neuroglia tissue with clumps of ependymal cells that has a propensity to obstruct the aqueduct [11,12]. If suspected, fine-cut sagittal T2-weighted images are considered to be the most useful sequence to demonstrate the presence of an aqueductal web [13]. The lack of early symptom onset is hypothesized to be due to compensated CSF flow dynamics in a growing cranium until adolescence or later—at this point, the CSF production has significantly increased and the absorption pathways are unable to cope [14,15,16,17]. Regarding the initial compensatory mechanism, there are three postulated theories: firstly, the presence of a partially patent aqueduct allowing normal CSF passage; next, the patient already has pre-existing substitute CSF passageways to circumvent the excess fluid; and finally, alteration of CSF production in these patients [18,19]. Etiologies for this condition include congenital, neoplasm, previous CNS infection and subarachnoid hemorrhage [12,14,20]. Interestingly, a proportion of hydrocephalic children with Neurofibromatosis Type 1 have been observed to have aqueductal webs as well [21,22]. Despite the myriad of causative factors, affected patients may manifest initial subtle signs and symptoms that only become prominent with ventricular dilatation from stenosis in adolescence or later [23]. For our patient, it remains uncertain if her diagnosis of early childhood septicemia is a contributing cause to her aqueductal web. We postulate that systemic infection during childhood may have previously affected her CNS, causing multiple intraventricular adhesions and ependymal scars. Nonetheless, we acknowledge that this hypothesis is unproven and continued follow-up is necessary to ensure the patient’s well-being.

### 3.2. Role of Neuroendoscopy for Aqueductal Web-Related Hydrocephalus: Technical Pitfalls

In contemporary neurosurgery, ETV is an efficacious procedure for hydrocephalus secondary to an obstructive etiology. This minimally invasive CSF diversion procedure has gained popularity because of its role in avoiding complications associated with childhood VPS implants [8]. Most ETV complications are directly related to the procedure itself [24]. Morbidity rates are cited to be as high as 31.2% and overall mortality rates range between 0.28% and 1.28% [24,25,26]. Structural injuries include trauma to blood vessels and cortical tissue at the puncture site leading to subdural hemorrhage, basilar artery perforation, oculomotor palsy and thalamic damage [27,28]. During ETV surgery, the standard technique of creating a stoma is by blunt perforation of the floor of the third ventricle. For most patients, the floor of the third ventricle is often thinned out and transparent. Under such circumstances, the ETV procedure is straightforward because the underlying neurovascular structures can be identified [28]. However, there are selected cases (as observed in our patient) whereby the floor is thick and opaque—a common consequence of previous infection and/or bleeding [24,28]. Here, the thickened floor may obscure the underlying brainstem vessels and nerves [28]. Thus, attempting to advance through such membranes can lead to unnecessary bleeding and stretching of surrounding critical structures [24]. Pertaining to the management of the web, there is presently no consensus. Although aqueductoplasty to open the blockage has been described as a treatment modality for these patients, there are others who advocate for ETV instead [4,11,22]. This is because the former technique has been observed to have a high risk of failure during long-term follow-up [11,23]. Nevertheless, the option of aqueductoplasty may be reserved for a subset of patients in whom ETV is not feasible but should be combined with stenting to avoid reclosure of the aqueduct [23].

### 3.3. Hyponatremia Following ETV Surgery: An Outline

Separately, symptomatic hyponatremia following ETV surgery is rare [29,30,31,32,33,34]. Table 2 summarizes a list of published studies that report hyponatremia after ETV based on a targeted literature review.

Hyponatremia is a common electrolyte imbalance encountered in patients with neurological diseases [35,36]. Under normal circumstances, the antidiuretic hormone (ADH; also known as arginine vasopressin or AVP) responds to an increase in serum osmolality to retain water in the renal nephrons. In contrast, patients with SIADH have unregulated secretion of vasopressin despite hypotonicity of the serum. Axiomatically, water intake combined with a high concentration of ADH leads to antidiuresis eventually resulting in hyponatremia [35]. To date, the diagnosis of SIADH has usually relied on a combination of essential and supporting criteria [35,37] (Table 3).

Based on our patient’s symptoms, laboratory investigations and treatment response, we acknowledge that her clinical presentation was a mixed picture. Nonetheless, there are pertinent factors as demonstrated in Table 3 that favored a working diagnosis of hyponatremia secondary to SIADH. This is because we are aware that despite an increased urinary sodium excretion expected in patients with SIADH, there are selected cases whereby its absence does not rule out the diagnosis. Similarly, the presence of an isolated high urinary sodium cannot confirm the diagnosis of SIADH [35]. Conversely, some patients with SIADH may have reduced urinary sodium if they become hypovolemic or solute-depleted, especially in cases of dehydration or solute depletion. Regarding the patient’s slightly raised TSH levels, we believe that this is likely a transient event in view of her recent surgery, normal free T4 results and clinically euthyroid status [38,39].

Separately, from a neurosurgical perspective, SIADH is often recognized to be a consequence of untreated hydrocephalus whereby there is ongoing mechanical pressure against the ventricles due to CSF volume buildup [40,41]. Here, the expansion of the third ventricle causes local compression on the supraoptic and paraventricular nuclei of the hypothalamus [41]. This direct pressure on the hypothalamus then causes release of ADH despite normal plasma osmolality, resulting in hyponatremia due to SIADH [40,42,43]. Therefore, the resolution of hydrocephalus via CSF diversion alleviates pressure on the hypothalamus, which reduces the release of ADH leading to resolution of serum hypoosmolality and serum hyponatremia [40]. Of note, this theory comes with a caveat—there is no pre-clinical study or animal model that has proven this observation [40]. Furthermore, most reported cases of post-ETV serum sodium imbalance are linked to diabetes insipidus (DI)—a consequence of iatrogenic damage to the hypothalamic–pituitary apparatus during surgery [29]. Other causative factors that cause electrolyte anomalies cited in the literature include the hypothalamic centers undergoing momentary irritation and/or traction during active fenestration of the third ventricle floor and choice of irrigation fluid [34,44,45].

Specifically, the pathogenesis of SIADH after ETV remains unelucidated. Here, its occurrence has been theorized to be caused either by surgical disruption of the supra-optico-hypophyseal tract or injury to the sub-forniceal organ [29,33,46]. Although it is uncertain why SIADH had a delayed onset in our patient, the timing of its occurrence is similar to other post-ETV cases previously reported in the literature [32,33]. Separately, Lang et al. previously proposed the use of neuro-navigation to plan an optimal tract trajectory to avoid this complication by reducing sub-forniceal manipulation [29]. Despite the use of stereotaxy for surgery, our patient still developed post-operative hyponatremia. Regardless, clinicians should be mindful that acute hyponatremia after neurosurgical procedures is a potentially life-threatening event that demands prompt recognition [47]. A thorough clinical assessment of the patient’s volume status and laboratory tests that include blood and urine electrolytes, serum and urine osmolality, the thyroid, the kidney, adrenocortical functions, and uric acid are recommended [48].

At this juncture, the importance of understanding sodium pathophysiology to facilitate correct management in a neurosurgical patient should be prioritized [49]. The contemporary literature cites that hyponatremia due to SIADH or cerebral salt wasting (CSW) is the most commonly encountered sodium imbalance scenario in neurosurgery [49,50]. Initially described in 1950, CSW is defined as a renal loss of sodium during intracranial disease leading to hyponatremia and a concurrent decrease in extracellular fluid volume [51]. This less common condition is typically associated with a myriad of intracranial pathologies, especially subarachnoid hemorrhage secondary to aneurysmal rupture and traumatic brain injury [49,52,53]. Various natriuretic factors such as brain natriuretic peptide (BNP) are believed to contribute to CSW. However, its exact pathophysiology is not yet completely elucidated [51]. Also, some experts believe that the diuresis and natriuresis observed in CSW is a simply a consequence of antidiuresis following SIADH [49,54].

From a clinical perspective, distinguishing between SIADH and CSW is crucial due to the divergent nature of treatment for each diagnosis [51]. Typically, SIADH patients have a high plasma volume with low serum osmolality, whereas CSW is characterized by a low plasma volume, dehydration, low serum osmolality, and a high urinary sodium excretion [51]. Hence, SIADH requires fluid restriction as vasopressin is the cause of relative water excess whereas conscientious salt replacement to compensate renal salt wasting using either isotonic or hypertonic saline is needed for CSW [51,55]. Although our patient was neither clinically dehydrated nor had a low serum osmolality, we were cautious to ensure that the initial treatment administered (i.e., trial of fluid restriction) was closely supervised by our pediatric endocrinologist.

Following that, the diagnosis of DI should be mentioned to complete the discussion on sodium imbalances related to ETV. This is because transient DI post-ETV is a recognized but infrequent complication [56]. Broadly speaking, central DI is caused by decreased secretion of antidiuretic hormone (ADH) which is produced by the hypothalamic neurons in the supraoptic and paraventricular nuclei [57]. Here, central DI is postulated to be caused by damage to the magnocellular neurons of the hypothalamus during the fenestration of the floor of the third ventricle. Clinically, the patient presents with loss of large volumes of diluted urine in the presence of hypernatremia and normal or high serum osmolality. Management focuses on a combination of ensuring adequate hydration and replacing the ADH deficit with desmopressin (DDAVP) under close supervision of an experienced pediatric endocrinology team.

## 4. Conclusions

We herein report a case of delayed onset of obstructive hydrocephalus secondary to an aqueductal web, the treatment challenges faced and the patient’s unexpected occurrence of post-operative hyponatremia. Key emphases are on perioperative sodium evaluation and the technical ETV nuances required to navigate a thickened third ventricular floor whilst simultaneously avoiding hypothalamic injury. We acknowledge the limitations of our single-patient experience and the paucity of similar studies. Nevertheless, we hope that our case can contribute to the current body of information on this topic, especially pertaining to the importance of emergent biochemical investigations to initiate the correct treatment.

This learning case necessitates the need for clinicians to be mindful of subtle symptoms in a patient whose clinical signs may not be straightforward. Here, the role of pre-operative counseling and close rapport with caregivers should be prioritized. As the way forward, we advocate continued research efforts in pediatric hydrocephalus for better disease understanding and management for affected children.

## Figures and Tables

**Table 1:** Summary of relevant investigations performed (abnormal results highlighted in bold italics). (Abbreviations: AM = ante meridiem; PM = post meridiem; ND = not done; POD = post-operative day).

Investigation Performed ^1^	Value (Normal Range with Units)	Before Surgery	POD 6	POD 6 (Repeated)	POD 7 (AM)	POD 7 (PM)	POD 8	POD 9	POD 16	POD 26
Sodium, serum	138 to 145 mmol/L	141	***121***	***122***	***122***	***132***	***130***	***134***	140	141
Potassium, serum	3.4 to 4.7 mmol/L	4.2	4.6	4.7	4.3	4.1	4.3	4.2	4.2	4.4
Urea, serum	2.6 to 6.8 mmol/L	3.7	3.5	3.3	3.4	4.0	4.5	4.5	4.5	3.9
Creatinine, serum	60 to 72 μmol/L	47	34	***31***	37	41	53	42	53	56
Osmolality, serum	275 to 300 mOsm/kg	ND	ND	***253***	ND	ND	ND	ND	ND	ND
Osmolality, urine	50 to 1200 mOsm/kg	ND	ND	210	ND	ND	ND	ND	ND	ND
Sodium, urine	<20 mmol/L	ND	ND	<20	ND	ND	ND	ND	ND	ND
Thyroxine (T4) free, serum	10 to 25.7 ρmol/L	ND	ND	14.6	ND	ND	ND	ND	ND	ND
Thyroid stimulating hormone (TSH), serum	0.5 to 4.5 mIU/L	ND	ND	***7.84***	ND	ND	ND	ND	ND	ND

^1^ Serum cortisol not done as patient was still on a low dose of oral dexamethasone at this time.

**Table 2:** Focused list of studies that report hyponatremia after ETV. (Abbreviations: N.A. = not applicable; POD = post-operative day; SIADH = syndrome of inappropriate antidiuretic hormone).

S/N	Year/Authors	Etiology of Hydrocephalus	Number of Patients (%)	Cause of Hyponatremia	Timing of SIADH Diagnosis
1	2000/Vaicys & Fried [33]	Post-traumatic hydrocephalus	1 patient	SIADH	POD 4
2	2005/Baykan et al. [34]	Not mentioned	5 out of 210 patients (2.4%)	Not mentioned	N.A.
3	2012/Lang et al. [29]	Not mentioned	5 out of 32 patients (15.6%)	Not mentioned ^1^	N.A.
4	2014/Shigeeda et al. [32]	Posterior fossa tumor	1 patient	SIADH	POD 6
5	2016/Kulkarni et al. [31]	Not mentioned	13 out of 336 (3.6%)	Not mentioned	N.A.
6	2020/Furtado et al. [30]	Myelomeningocele	1 out of 43 patients (2.3%)	Not mentioned	N.A.
7	2025/our case	Aqueductal web	1 patient	SIADH	POD 6

^1^ Postulated to be either SIADH or cerebral salt wasting.

**Table 3:** Criteria for SIADH diagnosis. (Adapted from [35,37]).

Essential	Our Patient
Serum sodium < 135 mmol/L	✓
Serum osmolality < 275 mOsm/kg	✓
Urine osmolality > 100 mOsm/kg	✓
Clinical euvolemia ^1^	✓
Urine sodium > 30 mmol/L (normal dietary salt and fluid intake)	✕
Normal thyroid and adrenal function	Equivocal (clinically euthyroid)
No concurrent use of diuretics	✕
**Supporting**	
Plasma uric acid < 0.24 mmol/L	Not done
Blood urea nitrogen < 3.6 mmol/L	✓
Fractional sodium excretion > 1%; fractional urea excretion > 55%	Not done
Improvement of hyponatremia with fluid restriction	✓
Failure to improve hyponatremia after 0.9% normal saline infusion	Not done

^1^ Patient did not have profound diuresis or hypotension during inpatient stay.

## Data Availability

The original contributions presented in this study are included in the article. Further inquiries can be directed to the corresponding author.
